# Burden and risk profile of acute kidney injury in severe COVID-19 pneumonia admissions: a Finding from Jimma University medical center, Ethiopia

**DOI:** 10.1186/s12882-024-03522-0

**Published:** 2024-03-20

**Authors:** Ebrahim Kelil Umer, Abel Tezera Abebe, Yabets Tesfaye Kebede, Nardos Tilahun Bekele

**Affiliations:** 1 Department of Internal Medicine, Adama Hospital Medical College, Adama, Ethiopia; 2 School of Medicine, Faculty of Medical Sciences, Institute of Health, Jimma University, Jimma, Ethiopia; 3 Department of Public Health, Adama Hospital Medical College, Adama, Ethiopia

**Keywords:** Acute kidney injury, COVID-19, Ethiopia

## Abstract

**Background:**

Acute kidney injury (AKI) is a serious complication of the Corona Virus Disease of 2019 (COVID-19). However, data on its magnitude and risk factors among hospitalized patients in Ethiopia is limited. This study aimed to determine the magnitude of AKI and associated factors among patients admitted for severe COVID-19 pneumonia.

**Methods:**

An institution-based retrospective cross-sectional study was conducted among 224 patients admitted to Jimma University Medical Center in Ethiopia for severe COVID-19 pneumonia from May 2020 to December 2021. Systematic random sampling was used to select study participants. Medical records were reviewed to extract sociodemographic, clinical, laboratory, therapeutic, and comorbidity data. Bivariable and multivariable logistic regressions were performed to examine factors associated with AKI. The magnitude of the association between the explanatory variables and AKI was estimated using an adjusted odds ratio (AOR) with a 95% confidence interval (CI), and significance was declared at a *p*-value of 0.05.

**Results:**

The magnitude of AKI was 42% (95% CI: 35.3–48.2%) in the study area. Mechanical ventilation, vasopressors, and antibiotics were required in 32.6, 3.7, and 97.7% of the patients, respectively. After adjusting for possible confounders, male sex (AOR 2.79, 95% CI: 1.3–6.5), fever (AOR 6.5, 95% CI: 2.7–15.6), hypoxemia (AOR 5.1, 95% CI: 1.4–18.9), comorbidities (AOR 2.8, 95% CI: 1.1–7.0), and severe anemia (AOR 10, 95% CI: 1.7–65.7) remained significantly associated with higher odds of AKI.

**Conclusion:**

The burden of AKI among patients with severe COVID-19 pneumonia is high in our setting. Male sex, abnormal vital signs, chronic conditions, and anemia can identify individuals at increased risk and require close monitoring and prevention efforts.

## Introduction

COVID-19 has affected millions of people worldwide with significant mortality and morbidity, along with a catastrophic economic and social burden [1, 2]. Although respiratory failure and diffuse alveolar injury were the two most obvious features, other organs, particularly the kidneys, were also involved [3].

Patients admitted with severe COVID-19 pneumonia often develop AKI, which confers an increased risk of mortality [2, 4]. Globally, reported estimates of the incidence of AKI in COVID-19 hospitalized patients vary in different parts of the world [5, 6]. Studies from China have found a relatively low incidence of AKI, ranging from 4.7 to 29% among hospitalized COVID-19 patients [7]. However, research from the United States and Brazil has revealed a markedly higher incidence, with 37 to 57% of hospitalized COVID-19 patients experiencing AKI [8, 9]. Older age, diabetes, hypertension, and the need for mechanical ventilation are documented risk factors for AKI in COVID-19 [10–12].

However, data on AKI incidence and risk factors among hospitalized patients in Ethiopia is lacking, despite its tremendous burden on patients and health systems [13, 14]. Lack of renal replacement therapy, late patient presentation, and limited critical care capacity worsen the outcomes of AKI [15]. This knowledge gap delays the recognition of AKI, appropriate management, and avoidable progression to chronic kidney disease (CKD) in COVID-19 patients. Therefore, understanding AKI’s local epidemiology and risk factors is imperative to guide prevention, earlier recognition, and optimal treatment.

The aims of this study are twofold. First, it seeks to determine the magnitude of AKI among patients admitted for severe COVID-19 pneumonia. Estimating the scale of AKI in this high-risk population will identify the burden on patients and hospital resources. Second, it aims to identify factors associated with AKI, including variables such as sociodemographic, clinical, laboratory, treatment, and comorbidities. Understanding the correlates of AKI can guide screening and prevention efforts to reduce kidney complications in COVID-19 patients. Furthermore, it will aid other researchers in evaluating the national burden of AKI in COVID-19 patients.

## Conceptual framework

**Fig. 1:**
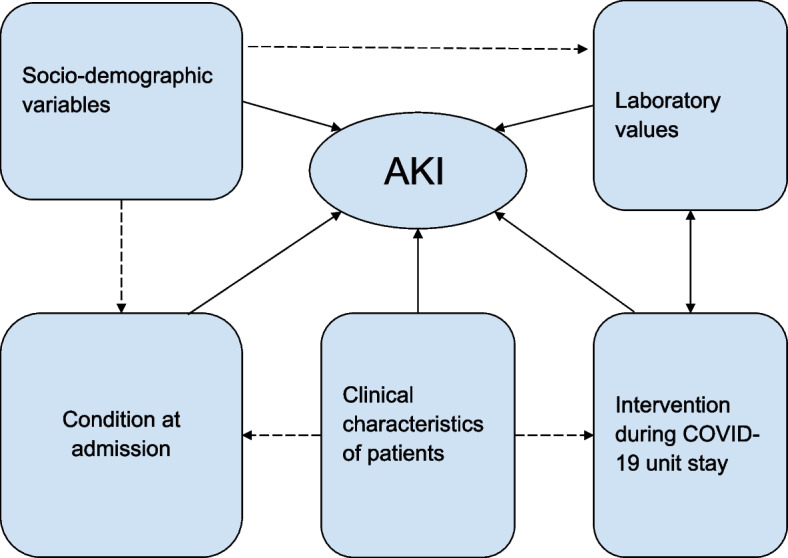
Conceptual framework adapted after review of different literature on factors associated with AKI in COVID-19 patients [16–18]

A conceptual framework, derived from an extensive review of various literature sources discussing the factors linked to Acute Kidney Injury (AKI) in patients with COVID-19, is presented in Fig. 1.

## Methods

### Study design, area, and period

This institutional-based retrospective cross-sectional study was conducted at Jimma University Medical Center (JUMC) from May 1, 2020, to December 30, 2021. JUMC is located in Jimma town, 352 km southwest of Addis Ababa, the capital of Ethiopia. Since 1964, JUMC has been the only referral hospital in southwest Ethiopia. It serves as a referral point for more than 15 million people in various regions and neighboring countries. The hospital provides services to 15 million people. During the COVID-19 pandemic, the hospital established a special COVID-19 treatment unit to handle disease emergencies. We conducted the study on patients who were admitted to this dedicated COVID-19 unit.

### Population

The source population comprised all COVID-19 patients admitted to the COVID-19 unit at JUMC. The study population included all COVID-19 patients admitted to the JUMC COVID-19 unit from May 1, 2020, to December 30, 2021.

### Inclusion criteria

All adult patients greater than 14 years of age who had been admitted to the JUMC COVID-19 unit and had a confirmed diagnosis of COVID-19 during the study period were included in the study.

### Exclusion criteria

Patients with incomplete medical records and a diagnosis of CKD at any stage before admission were excluded from the study. Furthermore, patients with known AKI causes such as pyelonephritis, inadequate renal perfusion, renal calculi, and other post-renal causes were excluded from the study.

### Sample size determination and sampling procedure

An independent sample size was calculated for the two specific objectives of the study, and the largest sample was taken. The sample size (*n*) was determined using a single population proportion formula with the assumptions of a 95% confidence level (*Z* = 1.96), a 5% margin of error (*d* = 0.05), and a 50% expected prevalence of AKI (*P* = 0.5). *n (i)* is the initial sample size.

$$n(i)=\frac{Z^{2}\cdot P\cdot \left(1-P\right)}{d^{2}}$$

$$n \left(i\right) = \frac{\left(\mathit{1.96}\right)^{\mathit{2}} \cdot \mathit{0.5}. \left(\mathit{1-0.5}\right)} {\mathit{0.05}^{\mathit{2}}} =\mathit{384}$$

Given that the source population is less than 10,000, specifically 542 individuals, applying the finite population correction formula is considered appropriate.

$$n=\frac{n(i)}{\mathit1+\frac{n(i)}N}=\frac{\mathit{384}}{\mathit1\mathit+\frac{\mathit{384}}{\mathit{542}}}\mathit=\mathit{225}$$

Adding a 5% non-response rate, the final sample size was 237 patients. The initial sample was obtained through simple random sampling. Subsequently, a systematic random sampling technique was applied to select participants from the COVID-19 unit registry book who met the inclusion criteria, with every *K* ^*th*^ participant included in the study. *K* was determined by the formula: $K=\frac Nn=\frac{\mathit{542}}{\mathit{225}}\mathit=\mathit2$, where *N* represents the population size and *n* is the desired sample size.

### Data collection tool and procedure

The data for this study was collected by medical interns who received training in data extraction using a pre-tested collection tool. The tool was designed to extract specific variables related to patient socio-demographics, admission details, clinical characteristics, medical interventions, laboratory test results, and comorbidities. The trained data collectors thoroughly reviewed the medical records and extracted pertinent data. The data collection process was supervised by medical residents to ensure accuracy and completeness.

### Data quality management

The questionnaire was pretested by administering it to interns and nurses working at JUMC to assess the quality of the data collection tool and improve it as needed. Data collectors were trained to have a common understanding of the data collection tools and to improve data quality.

### Variables

The dependent variable was AKI, defined using the Kidney Disease Improving Global Outcome (KDIGO) guideline [19].

The independent variables include:

- **Socio-demographic variables**: age, sex, residency, and source of admission
- **Patient condition upon admission**: the presence of comorbid illness, types of comorbidities, vital signs on admission (blood pressure, pulse rate, respiratory rate, temperature, oxygen saturation), nephrotoxic drug use before COVID-19 diagnosis, Glasgow coma scale (GCS) and the onset of symptoms in days
- **Laboratory values and imaging:** serum creatinine, serum potassium, white blood cell (WBC) count, random blood sugar (RBS), hemoglobin (Hgb), polymerase chain reaction (PCR), serum urea, high-resolution computed tomography (HRCT)
- **Intervention during COVID unit stay**: vasopressors use, antibiotics, and mechanical ventilation

### Operational definitions

**Acute Kidney Injury:** according to KDIGO guidelines, **AKI** is defined as an increase in serum creatinine of 0.3 mg/dl or more within 48 hours of observation**,** 1.5 times baseline or greater, which is known or presumed to have occurred within 7 days, or a reduction in urine volume below 0.5 ml/kg/h for 6 hours [19]. However, because we obtained secondary data and were unable to locate the documented urine output, we could only use the serum creatinine as a measuring unit for this investigation.

**A confirmed case of COVID-19:** is defined as testing positive for the SARS-CoV-2 virus via a reverse transcription polymerase chain reaction (RT-PCR) assay of a nasopharyngeal swab specimen.

**Date of onset of symptoms:** is the number of days that a patient had illness symptoms before being admitted.

**Previous use of nephrotoxic drugs:** refers to use within 2 weeks of admission to the COVID-19 treatment unit.

**Comorbidity:** refers to co-existing ailments that a patient has in addition to COVID-19 such as hypertension, diabetes mellitus, or congestive heart failure [20].

**Hypertension (HTN):** the current definition of HTN is systolic blood pressure (SBP) of 130 mmHg or higher and/or diastolic blood pressure (DBP) of more than 80 mmHg [21].

**Diabetes mellitus (DM):** a fasting blood sugar (FBS) level ≥ 126 mg/dl (7.0 mmol/l) or a random blood sugar (RBS) level ≥ 200 mg/dl (11.1 mmol/l) with symptoms is indicative of a diabetes diagnosis [22].

**Anemia:** is defined as a decrease in the percentage of red blood cells [23].

### Data processing and analysis

The collected data was entered into EPI Info version 7.1 and then transported to SPSS version 26 for analysis. Descriptive statistics, including frequencies, means, and medians, were used to provide a general description of the data. Binary logistic regression was used to model the relationship between the dependent variable and the independent variable. The statistical assumptions for binary logistic regression (adequacy of sample in each cross-tabulated result, expected count in each cell) were assessed, and multicollinearity was checked using the variation inflation factor (VIF) at VIF > 10, indicating the presence of multicollinearity. Simple logistic regression analysis was used to identify independent variables with a *P*-value of < 0.25 that were considered candidates for multiple logistic regression analysis. Multiple logistic regression was applied to estimate the effects of independent variables on AKI after adjusting for possible confounding effects. The regression model was fitted using the standard model-building approach. In the process of fitting the model, variables that didn’t have a significant association with AKI at a *p*-value < 0.05 were excluded from the model. The odds of AKI were estimated using an adjusted odds ratio with 95% confidence intervals. At this level, the significance of associations was declared at a *p*-value of 0.05. The model fitness test was checked by the Hosmer and Lemeshow goodness of fit test at a *p*-value ≥ 0.05.

## Results

### Socio-demographic characteristics and admission circumstances

In this study, 224 patient records were included, representing a 94.5% response rate based on the calculated sample size of 237. The median (IQR) age of patients was 60 years (45–70 years). Males comprised 147 (65.6%) of the total respondents (see Table 1).

**Table 1:** Sociodemographic characteristics of severe COVID-19 pneumonia patients admitted to Jimma University Medical Center, Jimma, Oromia, Ethiopia

Variables	Category	Frequency	Percent
**Age in years**	18–64	135	60.3
	65–85	83	37.1
	> = 85	6	2.7
**Sex**	Male	147	65.6
	Female	77	34.4
**Place of residence**	Urban	115	51.3
	Rural	109	48.7
**Source of admission**	Emergency OPD	137	61.2
	Medical ward	75	33.5
	Surgical ward	12	5.4

*OPD* outpatient department

### Patient’s condition upon admission

Patients’ clinical characteristics upon admission revealed that 60 (26.8%) were hypertensive, 79 (35.3%) had tachycardia, and 218 (97.3%) were tachypneic. Of the patients, 74 (33%) were febrile, and 132 (58.9%) had normal GCS. 141 (62.9%) of study participants had a comorbid illness, with diabetes accounting for 47 (21%). Notably, 134 (60.9%) patients experienced shortness of breath as their first symptom, and 85 (37.9%) reported symptom onset within 4–5 days (see Table 2).

**Table 2:** Patient’s condition upon admission of severe COVID-19 pneumonia patients admitted to Jimma University Medical Center, Jimma, Oromia, Ethiopia

Variable	Category	Frequency	Percent
**Blood pressure**	Normal (90/60–120/80 mmHg)	91	40.6
	Elevated (130/90–140/90 mmHg)	67	29.9
	Hypertension (> 140/90 mmHg)	60	26.8
	Hypotension (< 90/60 mmHg)	6	2.7
**Pulse rate**	Normal (60–100 beats per minute)	143	63.8
	Bradycardia (< 60 beats per minute)	2	0.9
	Tachycardia (> 100 beats per minute)	79	35.3
**Respiratory rate**	Normal (12–20 breaths per minute)	6	2.7
	Tachypnea (> 20 breaths per minute)	218	97.3
**Temperature (°C)**	Normal (35.5–37.5)	150	67
	Febrile (> 37.5)	74	33
**Oxygen saturation (SPO**_**2**_ **)**	Normal (> 93%)	34	15.2
	Hypoxemia (< 93%)	190	84.8
**GCS**	Normal (15/15)	132	58.9
	Lethargic (13–14/15)	66	29.5
	Stuporous (8–12/15)	26	11.6
**Comorbidity**	Yes	141	62.9
	No	83	37.1
**Types of Comorbidities**	Diabetes	47	21
	HIV	9	4
	Hypertension	60	26.8
	Heart failure	68	30.4
**Previous nephrotoxic drug use**	NSAID	12	5.4
	ACEI	33	14.7
	Other^a^	5	2.2
**Onset of symptoms in days**	< 4	51	22.8
	4–5	85	37.9
	5–7	66	29.5
	> 7	22	9.8

*GCS* Glasgow coma scale, *HIV* human immuno-deficiency virus, *NSAID* nonsteroidal anti-inflammatory drug, *ACEI* angiotensin-converting enzyme inhibitor

^a^Aminoglycoside, Chemotherapy

### Intervention during COVID-19 treatment unit stay

Out of the total patient population, 73 (32.6%) individuals required mechanical ventilation, and a substantial proportion, 215 (97.7%), received antibiotic treatment. Additionally, a smaller subset of 8 patients (3.7%) were administered a vasopressor.

### Laboratory values and imaging

Among the participants, 161 (72.5%) exhibited normal random blood sugar (RBS), while 117 (54.4%) had mild anemia. Elevated serum urea levels were observed in 167 (75.6%) patients upon admission, and 23 (10.7%) patients had high serum potassium levels. Leukocytosis was found in 98 (44.5%) patients. Furthermore, typical bilateral opacity is found in 145 (68.1%) patients under high-resolution computed tomography (HRCT) (see Table 3).

**Table 3:** Laboratory values and imaging of severe COVID-19 pneumonia patients admitted to Jimma University Medical Center, Jimma, Oromia, Ethiopia

Variable	Category	Frequency	Percent
**Random blood sugar**	Normal (70–180 mg/dl)	161	72.5
	Hyperglycemic (> 180 mg/dl)	61	27.5
**Hemoglobin (Hgb)**	Severe anemia (< 7 g/dl)	13	6
	Moderate anemia (7–10 g/dl)	13	6
	Mild anemia (10–12 g/dl)	117	54.4
	Normal (> 12 g/dl)	72	33.5
**Polymerase chain reaction (PCR)**	Positive	213	100
	Negative	0	0
**HRCT**	Typical bilateral GGO	145	68.1
	Other^a^	68	31.9
**Serum urea at admission**	Normal (5–20 mg/dl)	54	24.4
	High (> 20 mg/dl)	167	75.6
**Serum potassium**	Hypokalemia (< 3.5 mEq/l)	50	23.3
	Hyperkalemia (> 5 mEq/l)	23	10.7
	Normal (3.5–5.0 mEq/l)	142	66
**White blood cell count**	Leukopenia (< 5000 cells/mm^3^)	22	10
	Leukocytosis (> 10,000 cells/mm^3^)	98	44.5
	Normal (5000–10,000 cells/mm^3^)	100	45.5

*HRCT* high-resolution computed tomography, *GGO* ground glass opacity

^a^Normal imaging with positive PCR test or atypical imaging findings

### Magnitude of acute kidney injury

**Fig. 2:**
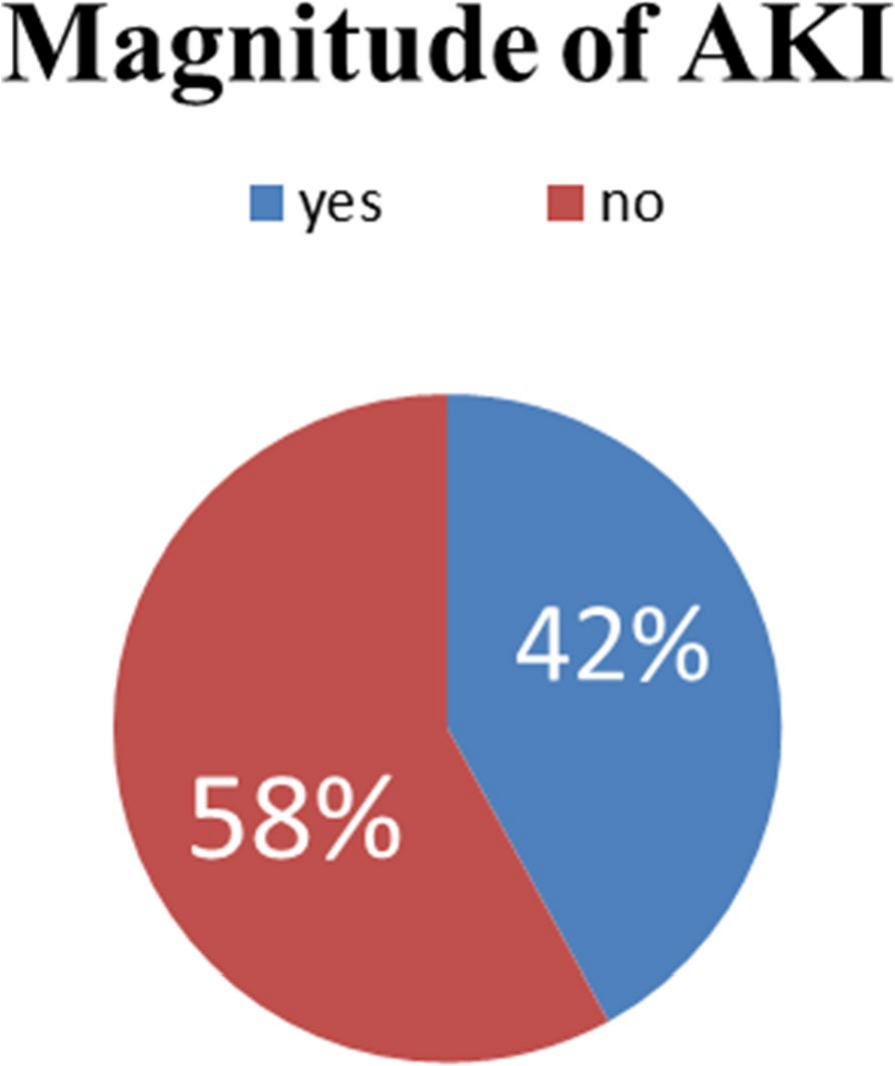
Magnitude of AKI among severe COVID-19 pneumonia patients admitted to Jimma University Medical Center, Jimma, Oromia, Ethiopia

The result of this study showed that the magnitude of acute kidney injury among severe COVID-19 pneumonia patients admitted to Jimma University Medical Center was 42% (95% CI: 35.3, 48.2). This is per the KDIGO-AKI definition (See Fig. 2).

### Factors associated with acute kidney injury

In the simple logistic regression analysis, several independent variables showed significant associations with AKI at a *p*-value < 0.25. These variables included age, sex, temperature, SPO2, GCS, comorbidity, hypertension, hemoglobin (Hgb), serum urea at admission, and white blood cell count (WBC). Subsequently, a multiple logistic regression model was applied, adjusting for all potential confounders. After this adjustment, only sex, temperature, SPO2, comorbidity, Hgb, and WBC remained significantly associated with AKI at a *p*-value < 0.05.

As per the results of the analysis, several factors exhibited significant associations with the odds of having AKI (Table 4):

1. Male patients had 2.79 times higher odds of developing AKI compared to females (AOR 2.79, 95% CI: 1.3, 6.5).
2. Febrile patients had 6.5 times higher odds of experiencing AKI (AOR = 6.5; 95% CI: 2.7, 15.6) in comparison to non-febrile patients.
3. Hypoxemic patients had 5.1 times higher odds of developing AKI (AOR = 5.1; 95% CI: 1.4, 18.9) than those with normal SPO2 levels.
4. Patients with comorbidities had 2.8 times higher odds of AKI (AOR = 2.8; 95% CI: 1.1, 7.0) in comparison to patients without comorbid conditions.
5. Patients with severe anemia had 10 times higher odds of AKI (AOR = 10; 95% CI: 1.7, 65.7) than those within the normal hemoglobin range.

**Table 4:** Factors associated with AKI among severe COVID-19 pneumonia patients admitted to Jimma University Medical Center, Jimma, Oromia, Ethiopia

Variable	AKI		COR (95%CI)	AOR (95%CI)
	Yes	No		
**Age**				
18–64	50(37)	85(63)	Ref	Ref
> =65	44(49.4)	45(50.6)	1.6(0.9, 2.8)^*^	1.6(0.7, 3.4)
**Sex**				
Female	26(34)	51(66)	Ref	Ref
Male	84(57.1)	63(42.9)	2.6(1.2, 2.3)^**^	2.79(1.3, 6.5)^*^
**Temperature**				
Normal	52(34.7)	98(65.3)	Ref	Ref
Febrile	42(56.8)	32(43.2)	2.4(1.3, 4.3)^**^	6.5(2.7, 15.6)^***^
**Oxygen Saturation (SPO2)**				
Normal	8(23.5)	26(76.5)	Ref	Ref
Hypoxemia	86(45.3)	104(54.7)	2.6(1.1, 6.2)^*^	5.1(1.4, 18.9)^*^
**GCS**				
Normal	46(34.8)	86(65.2)	Ref	Ref
Lethargic	33(50)	33(50)	1.8(1, 3.4)^*^	0.9(0.3, 2.5)
Stuporous	15(57.7)	11(42.3)	2.5(1,6) ^*^	0.2(0.05, 0.8)^*^
**Comorbidity**				
No	20(24.1)	63(75.9)	Ref	Ref
Yes	74(2.5)	67(47.5)	3.4(1.9, 6.3)^***^	2.8(1.1, 7.0)^**^
**Hypertension**				
No	59(36)	105(64)	Ref	Ref
Yes	35(58.3)	25(41.7)	2.4(1.3, 4.5)^**^	1.2(0.5, 3.2)
**Hemoglobin (Hgb)**				
Severe anemia	11(84.6)	2(15.4)	7.2(1.5, 35.2)^*^	10(1.7, 65.7)^*^
Moderate	10(76.9)	3(23.1)	4.4(1.1, 17.3)^*^	2.8(0.5, 15.2)
Mild	42(35.9)	75(64.1)	0.7(0.4, 1.3)	0.5(0.2, 1.2)
Normal	31(43.1)	41(56.9)	Ref	Ref
**WBC count**				
Normal	36(36.0)	64(64)	Ref	Ref
Leukopenia	20(90.9)	2(9.1)	17.7(3.9,80.4)^***^	68.3(9.8, 474.7)^***^
Leukocytosis	38(38.8)	60(61.2)	1.12(0.6,2)	1.2(.5, 2.6)

*GCS* Glasgow coma scale, *WBC* White blood cell count

* *P* < 0.05, ** *P* < 0.01, *** *P* < 0.001

## Discussion

This study aimed to determine the magnitude and factors associated with acute kidney injury (AKI) among 224 patients admitted for severe COVID-19 pneumonia in Ethiopia. The key findings and their implications are discussed below in the context of prior literature:

### Magnitude of AKI

The magnitude of AKI in our cohort was 42%, which is relatively high compared to other studies that found around 0.5–29% of hospitalized COVID-19 patients developed AKI [5–7, 24]. However, our estimate is aligned with studies focused only on critically ill patients, of whom 38–45% developed AKI [8, 9, 11, 25]. The high burden in our study may be attributed to the inclusion of only severe pneumonia cases requiring hospitalization. It highlights the need for AKI prevention and early recognition in patients with serious COVID-19 to improve outcomes.

### Risk factors for AKI

We identified male sex, fever, hypoxemia, comorbidities, and severe anemia as factors significantly associated with a higher AKI risk in COVID-19 patients.

Male gender was significantly associated with increased AKI risk, which is consistent with research from China, Brazil, Switzerland, and Italy [26–31]. A possible explanation for the association with the male sex is hormonal differences affecting kidney injury response [32]. Furthermore, higher ACE2 (Angiotensin Converting Enzyme 2) and TMPRSS2 (Type II Transmembrane Serine Protease) expression in male tissue than in female tissue suggests their increased susceptibility to direct damage by the SARS-COV-2 virus [33–35].

Fever and hypoxemia may reflect more severe systemic inflammation from COVID-19 that predisposes to organ dysfunction. COVID-19 patients with fever had a higher likelihood of experiencing AKI, possibly due to concomitant sepsis and potential nephrotoxic drug use [36]. Hypoxemic patients also had increased odds of AKI compared to non-hypoxemic patients. The kidney’s vulnerability to hypoxia and related insults is attributed to the relatively low oxygen concentration in kidney tissues, particularly in the renal medulla, coupled with the high oxygen demand [37–39]. A study conducted in the metropolitan area of New York revealed significant disparities in the incidence of AKI between ventilated and non-ventilated patients. Among patients receiving ventilator support, the rate of AKI was notably higher at 89.7%, in contrast to 21.7% among those not on ventilators. Moreover, an overwhelming majority of patients (96.8%) requiring renal replacement therapy were found to be among those receiving ventilator support. These results underscore the association between the occurrence of AKI and mechanical ventilation, and thus potential hypoxia [3].

Our study revealed a significant association between AKI and the presence of comorbidities. This result is consistent with earlier meta-analyses, which also noted a strong association between the occurrence of AKI in COVID-19 patients and pre-existing conditions like diabetes and hypertension [40, 41]. Preexisting comorbid conditions like diabetes and hypertension impair vascular autoregulation, which worsens ischemic AKI [42, 43]. Anemia reduces renal oxygen delivery and has been linked to a higher risk of AKI in critically ill patients [44]. In patients with renal transplants, the prevalence is slightly elevated, ranging from 44 to 48% [45, 46].

These results are consistent with previous studies showing older males with comorbidities and indicators of greater COVID-19 severity are more prone to AKI [4]. Unlike prior research, we did not find age itself to be a significant risk factor when adjusted for other variables [2]. Our findings suggest these clinical parameters could help risk stratify patients needing closer AKI monitoring and optimization of modifiable factors like anemia.

### Treatment patterns

Invasive treatments like mechanical ventilation and vasopressors were required in one-third and less than 5% of patients, respectively. While we did not adjust for baseline illness severity, this likely reflects advanced COVID-19, where AKI develops more frequently. Over 95% of our cohort received antibiotics, though it is unclear what proportion was for primary COVID-19 treatment versus secondary infections. Prior work found that sepsis and nephrotoxic medications increased AKI rates in COVID-19 [3, 47]. Judicious use of life-saving but potentially kidney-harming therapies is warranted.

### Limitations

Our single-center design limits the generalizability of the findings. The retrospective nature meant relying on documentation for data extraction. We did not have access to urine output measurements, longitudinal creatinine values, or standard AKI staging. Our adjusted analysis could not account for all potential confounders, like medications and sequential organ failure assessment scores. Further prospective research with multivariate adjustment and AKI adjudication is needed to corroborate our results.

## Conclusions

This study found a high burden of AKI in patients hospitalized for severe COVID-19 pneumonia in Ethiopia. Male sex, abnormal vital signs, comorbidities, and anemia were identified as significant risk factors that could guide monitoring and prevention. These findings highlight the need for optimizing AKI management as part of COVID-19 treatment protocols. Further studies should explore the impacts of AKI on long-term kidney outcomes.

## Data Availability

Data will be shared upon request. abel.tezera.abebe@gmail.com (Abel Tezera Abebe, MD, Corresponding Author).
